# Early growth phase and caffeine content response to recent and projected increases in atmospheric carbon dioxide in coffee (*Coffea arabica* and *C. canephora*)

**DOI:** 10.1038/s41598-020-62818-x

**Published:** 2020-04-03

**Authors:** Fernando E. Vega, Lewis H. Ziska, Ann Simpkins, Francisco Infante, Aaron P. Davis, Joseph A. Rivera, Jinyoung Y. Barnaby, Julie Wolf

**Affiliations:** 1 Sustainable Perennial Crops Laboratory, U. S. Department of Agriculture, Agricultural Research Service, Beltsville, MD 20705 USA; 2 Adaptive Cropping Systems Laboratory, U. S. Department of Agriculture, Agricultural Research Service, Beltsville, MD 20705 USA; 3 El Colegio de la Frontera Sur (ECOSUR), Tapachula, Chiapas Mexico; 4 Royal Botanic Gardens, Kew, Richmond, Surrey UK; 5 Coffee Intelligence, LLC, Pasadena, CA 91105 USA; 6 Dale Bumpers National Rice Research Center, U. S. Department of Agriculture, Agricultural Research Service, Stuttgart, AR 72160 USA; 7 Present Address: Environmental Health Sciences, Mailman School of Public Health, Columbia University, New York, NY 10032 USA

**Keywords:** Climate change, Climate-change impacts, Projection and prediction

## Abstract

While [CO_2_] effects on growth and secondary chemistry are well characterized for annual plant species, little is known about perennials. Among perennials, production of *Coffea arabica* and *C. canephora* (robusta) have enormous economic importance worldwide. Three Arabica cultivars (Bourbon, Catimor, Typica) and robusta coffee were grown from germination to ca. 12 months at four CO_2_ concentrations: 300, 400, 500 or 600 ppm. There were significant increases in all leaf area and biomass markers in response to [CO_2_] with significant [CO_2_] by taxa differences beginning at 122–124 days after sowing (DAS). At 366–368 DAS, CO_2_ by cultivar variation in growth and biomass response among Arabica cultivars was not significant; however, significant trends in leaf area, branch number and total above-ground biomass were observed between Arabica and robusta. For caffeine concentration, there were significant differences in [CO_2_] response between Arabica and robusta. A reduction in caffeine in coffee leaves and seeds might result in decreased ability against deterrence, and consequently, an increase in pest pressure. We suggest that the interspecific differences observed (robusta vs. Arabica) may be due to differences in ploidy level (2n = 22 vs. 2n = 4x = 44). Differential quantitative and qualitative responses during early growth and development of Arabica and robusta may have already occurred with recent [CO_2_] increases, and such differences may be exacerbated, with production and quality consequences, as [CO_2_] continues to increase.

## Introduction

Because CO_2_ represents the sole source of carbon for photosynthesis, and because CO_2_ levels have been low for the recent geological past (<800,000 years before present), recent (317–412 ppm since 1960) and projected increases^1^ (450–600 ppm by 2050) represent a major shift in an essential resource needed for plant growth. The biological role of rising atmospheric carbon dioxide concentration [CO_2_] is well recognized as altering physical (e.g., growth rates, stomatal aperture), biochemical (e.g., carbon to nitrogen (C:N) ratios, photorespiration), phenological (e.g., time to flowering), and reproductive (e.g., seed yield) characteristics for a wide variety of plant taxa, including agricultural crops^2–6^.

Because interspecific and intraspecific variation exists in response to resource changes, there has been a merited focus on quantifying intraspecific variation that could be used as a means of selection for adaptation to rising [CO_2_] levels. For example, studies have confirmed that there is significant intraspecific variation in the yield response to future CO_2_ levels for cowpea (*Vigna unguiculata* (L.) Walp.)^7^; common bean (*Phaseolus vulgaris* L.)^8^, rice (*Oryza sativa* L.)^9–11^; wheat (*Triticum aestivum* L.)^12,13^ and soybean (*Glycine max* (L.) Merr.)^14^, such that breeders could begin to select for CO_2_ responsiveness among currently available germplasm.

However, such efforts have been focused, in general, to annual crops, particularly those of global importance (e.g., wheat, rice). Less attention, overall, has been given for CO_2_ selection among perennial crops. In that regard, coffee (*Coffea arabica* L. (Arabica coffee) and *C. canephora* Pierre ex A.Froehner (robusta coffee)) is one of the world’s most important perennial crops, and represents not only a widely traded agricultural commodity, but also a social and economic foundation for numerous tropical developing countries, with approximately 125 million people involved in coffee growing^15^. Although there are 124 *Coffea* species^16^, only two, Arabica and robusta are associated with the bulk of global coffee production^17^.

Arabica and robusta field responses to ca. 550 ppm CO_2_, with an emphasis on photosynthetic metabolism, is available^18^. Additional growth chamber studies evaluating temperature and [CO_2_] in the context of growth and photosynthetic acclimation response (including transformations in stomatal characteristics) are also available for coffee^19–22^. However, these data represent the growth and metabolic response of coffee following transfer of 12 to 18-month-old coffee plantlets into Free-Air CO_2_ enrichment (FACE) or [CO_2_] growth chambers. At present, any differential growth response within, or between Arabica and robusta to recent and projected increases in CO_2_ from germination through early growth (ca. 1 year) is not available. Yet, early exposure may be critical, as initial vegetative growth may represent the temporal period of greatest physiological sensitivity to additional CO_2_, for annuals^23^.

In addition to differential growth, there is substantial evidence that supplementary CO_2_ may reduce protein content and increase carbon to nitrogen (C:N) ratios for numerous plant taxa^4,24,25^ with potential effects on secondary compounds that have a high N content^26^. Caffeine (C_8_H_10_N_4_O_2_; 1,3,7-trimethylxanthine; ca. 29% N by molecular weight) may act as a defense against herbivores^27–29^ and consequently, CO_2_-induced changes in leaf and seed caffeine concentration may be of ecological interest^30^ including unforeseen consequences for climate change impact as a result of changes in plant-herbivore relationships^31^.

To determine the physiological impact of recent and projected increases in CO_2_ levels four *Coffea* taxa, i.e., three Arabica cultivars (Bourbon, Catimor, Typica) and robusta coffee, were grown from germination for approximately one year at CO_2_ concentrations of 300, 400, 500 or 600 ppm, and measured growth (plant height, leaf area, biomass, leaf weight, number of branches, dry weight), C: N ratio, and caffeine concentration (mg g^−1^).

## Results

**Figure 1:**
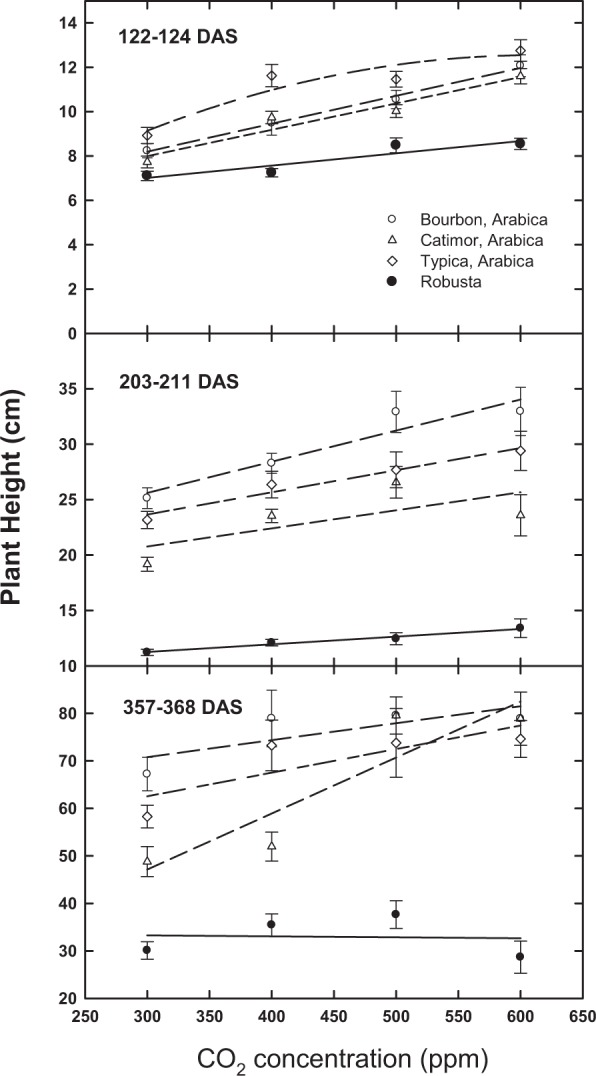
Change in plant height (Average + SE) as a function of days after sowing (DAS) and [CO_2_] for three Arabica cultivars and robusta coffee.

**Figure 2:**
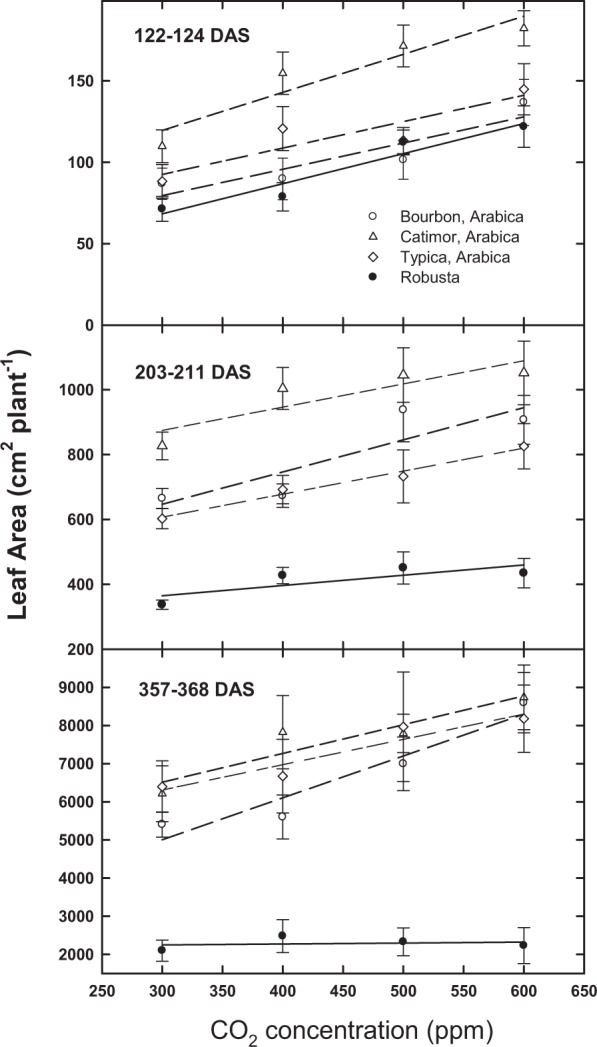
Change in leaf area (cm^2^ per plant, average + SE) for three Arabica cultivars and robusta coffee at three different sampling times (days after sowing, DAS) in response to [CO_2_].

Comparisons of plant height indicate significant increases at all sampling periods as a function of [CO_2_] above the 300 ppm baseline (Table 1; Fig. 1). However, by the 12-month period (357–368 days after sowing; DAS), there was no significant effect of [CO_2_] on plant height for robusta (Fig. 1). Similarly, [CO_2_] stimulation of leaf area was observed for all taxa at the 4 and 7-month period (122–124 and 203–211 DAS, respectively) in response to rising [CO_2_]; however, by the 12-month period, robusta plants had stopped responding (Fig. 2). Differences in leaf area as a function of [CO_2_] by Arabica/robusta were not significant (P = 0.20; Table 1).

**Table 1:** Statistical values for the three Arabica cultivars and robusta coffee response to recent and projected increases in atmospheric CO_2_ at three sampling periods (DAS, days after sowing).

Parameter	[CO_2_]	4 T	A/R	[CO_2_] × CV	[CO_2_] × A/R	[CO_2_] × 4 T
***122–124 DAS***						
Leaf Area	***	***	**	0.15	0.68	0.47
Abv. Ground Wt.	*	***	***	(*)	0.12	**
***211–213 DAS***						
Leaf Area	***	***	***	0.44	0.35	0.27
Abv. Ground Wt.	***	***	***	0.73	*	0.21
***366–368 DAS***						
% Nitrogen	***	0.12	*	0.30	0.66	0.38
C:N	***	**	***	0.30	0.37	0.27
Caffeine (mg g^−1^)	*	*	*	0.27	*	0.26
Height (cm)	***	***	***	0.82	0.43	0.53
True Leaf No.	0.07	***	***	0.99	0.63	0.91
Branch No.	***	***	***	0.27	*	*
Leaf Area	**	***	***	0.89	0.20	0.60
Leaf Wt.	**	***	***	0.93	(*)	0.42
Branch Wt.	**	***	***	0.98	0.54	0.93
Stem Wt.	**	***	***	0.73	0.16	0.34
Total Wt.	***	***	***	0.97	(*)	0.57

A/R is Arabica vs. robusta; [CO_2_] × CV is CO_2_ × Arabica cultivars only; [CO_2_] × 4 T is [CO_2_] × all four taxa. Total above ground weight and vegetative characteristics are in g per plant. Leaf area is in cm^2^. (*)Indicates a P value between 0.05 and 0.10; *Indicates a P value between 0.05 and 0.01; **Indicates a P value between 0.01 and 0.001; ***Indicates a P value < 0.001.

**Figure 3:**
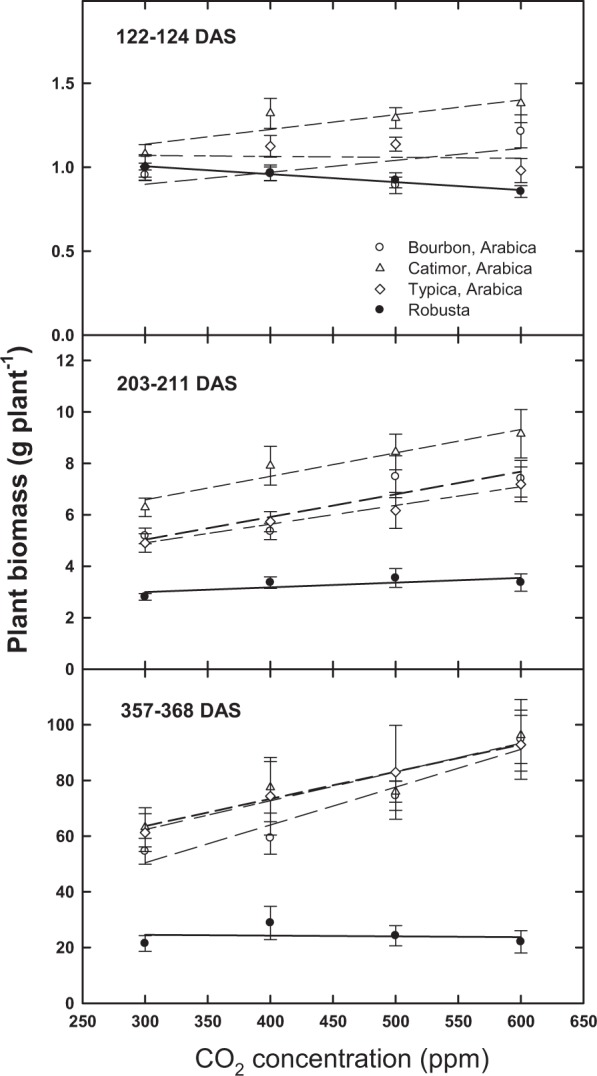
Change in total plant biomass (grams per plant, average + SE) for three Arabica cultivars and robusta coffee at three different sampling times (days after sowing, DAS) in response to [CO_2_].

**Figure 4:**
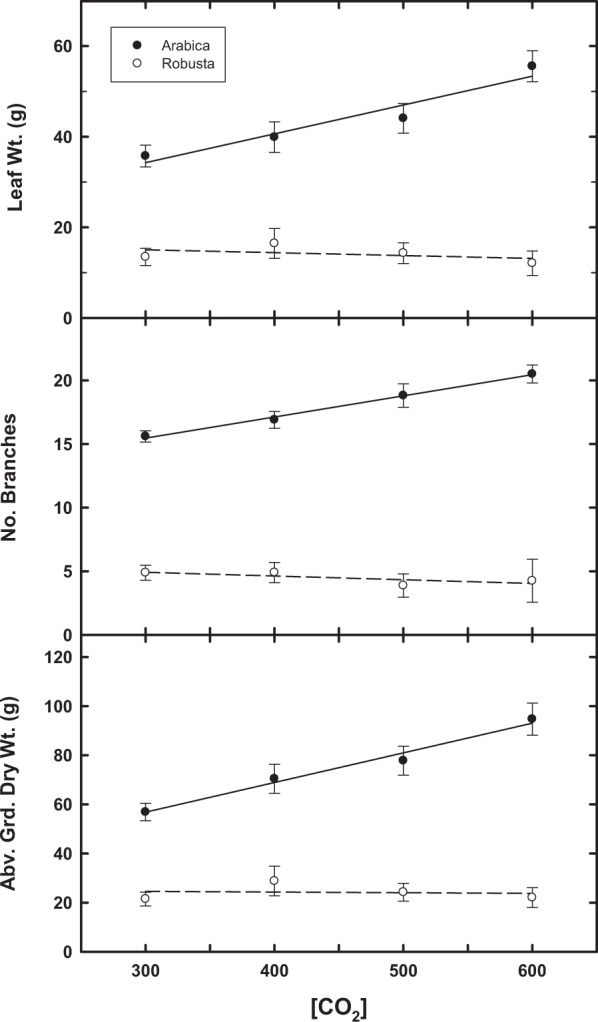
Differential changes between Arabica and robusta coffee (average + SE) for above ground dry weight, number of branches, and leaf weight in response to [CO_2_] at 357–368 DAS.

For above-ground plant biomass, increasing [CO_2_] resulted in [CO_2_] by Arabica cultivar responses at the 4-month period (122–124 DAS), but not at 7 (203–211 DAS) or 12 months (357–368) (Table 1; Fig. 3). By the end of the study (~12 months), no effect of [CO_2_] was evident for robusta (Fig. 3); however, marginally significant differences (P < 0.1) between Arabica and robusta for above ground dry weight were observed (Table 1; Fig. 4). Overall, by 12 months, Arabica, on average, showed a significant response to increasing [CO_2_] for several vegetative parameters; whereas robusta was insensitive to [CO_2_] for several vegetative parameters (Table 1, Fig. 4).

In addition to growth and vegetative response, [CO_2_] induced changes in qualitative parameters, e.g., % N, carbon to nitrogen (C:N) ratio and caffeine concentration are of interest.

**Figure 5:**
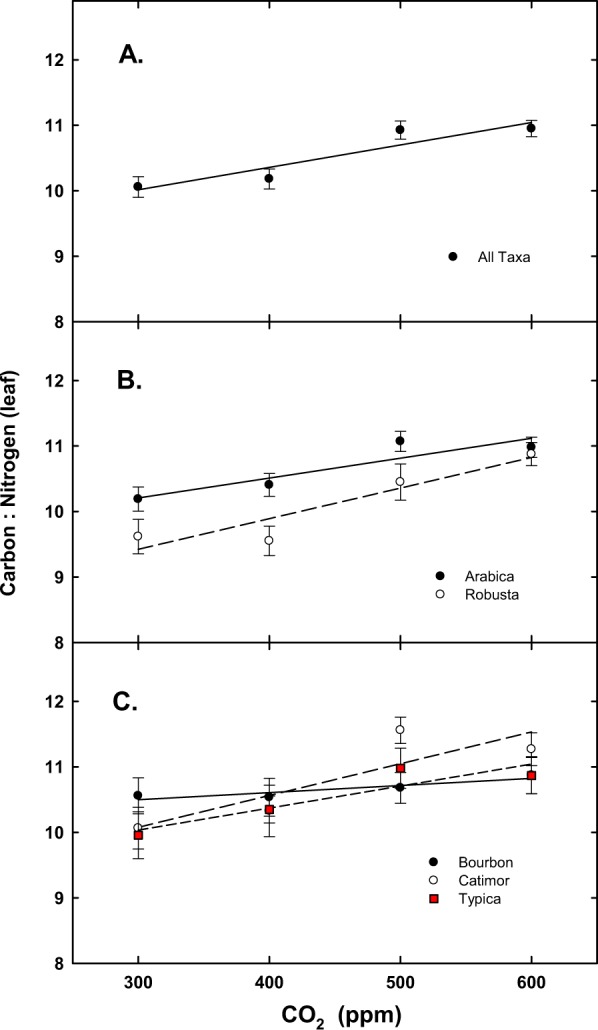
Response of carbon to nitrogen ratio (+SE) for: (**A**) all taxa; (**B**) Arabica and robusta coffee; and (**C**) all Arabica cultivars in response to [CO_2_].

**Figure 6:**
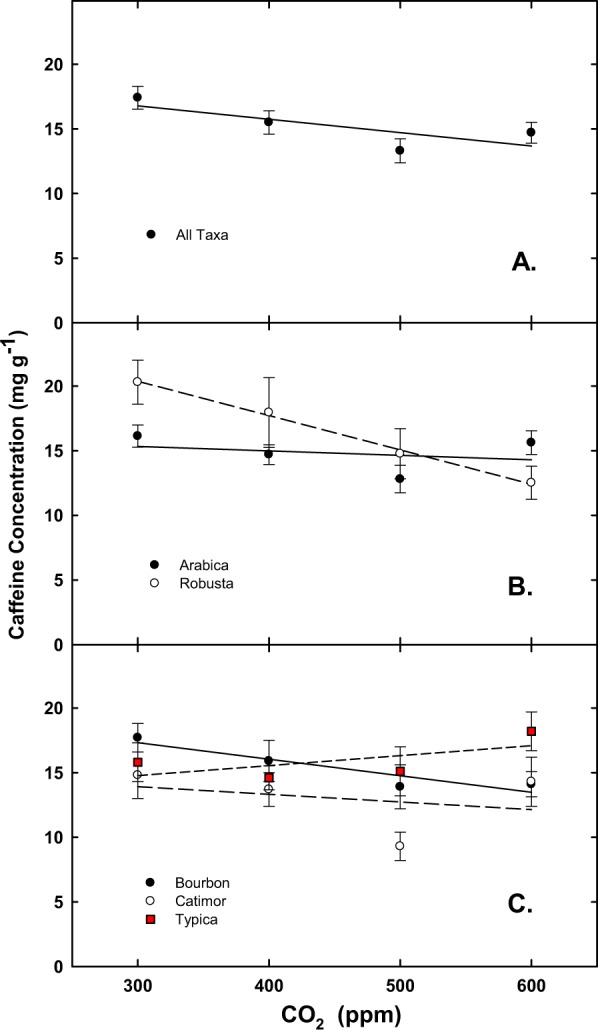
Caffeine concentration as a function of [CO_2_] (average +SE) for: (**A**) all taxa; (**B**) Arabica and robusta coffee; and, (**C**) all Arabica cultivars in response to [CO_2_].

For the final harvest, when averaged for all taxa, significant effects were noted for C:N ratio for [CO_2_] (Table 1, Fig. 5A), and for Arabica vs. robusta (Table 1, Fig. 5B). Differences for the Arabica cultivars were also noted for C:N and caffeine, but not for % N (P = 0.12) (Table 1; Fig. 5C). Interactions, [CO_2_] × Arabica cultivars only, Arabica vs robusta or cultivar (all four taxa) were not significant for % N or C:N ratio (Table 1). When averaged for all taxa, there were no significant differences in caffeine (Table 1, Fig. 6A), in contrast to a significant difference in reductions of caffeine with increasing [CO_2_] for robusta but not Arabica (Table 1, Fig. 6B). No caffeine concentration (mg g^−1^) differences were observed among the three Arabica cultivars (Table 1, Fig. 6C),

## Discussion

Plant growth and development, assuming physiologically relevant temperatures, relies on four environmental (abiotic) resources: nutrients (macro- and micro-), light, water, and CO_2_. Any change in one (or more) of these resources could lead to a change in fitness among different genotypes^32^. In managed plant systems, there have been numerous studies indicating intraspecific variation to [CO_2_] with respect to vegetative and physiological characteristics, including yield, for a given crop species^33^. Sufficient variation has been reported so that screening or selecting for enhanced [CO_2_] responsive cultivars offers a potential means to increase crop yields and improve nutrition, which are important steps to help adapt production to global climate change^2,13,33,34^.

Efforts have been made to identify variation in productivity responses to elevated [CO_2_] for forest tree species^35^. Such studies have found considerable intraspecific variation in photosynthesis, stem biomass and volume for poplar, pine, birch, eucalyptus, etc., at elevated [CO_2_], suggesting that under non-limiting environmental conditions, (e.g., temperature, nutrients, water), intraspecific variation could be used to select for increased productivity as atmospheric CO_2_ increases^36,37^. However, similar efforts for intraspecific or interspecific selection to [CO_2_] among tree crops (e.g., apples, cacao) are, at present, unavailable, despite experiments showing that trees can be more responsive than herbaceous plants to elevated CO_2_^34^.

In the current study, while Arabica cultivars showed a significant response to rising [CO_2_] above the mid-20^th^ century baseline (i.e., 317 ppm) for leaf area and growth parameters, significant variation among Arabica cultivars was not evident for any DAS harvest. In contrast, robusta coffee was consistently less responsive to rising [CO_2_] for growth biomass traits. Accordingly, there is a clear interspecific (between species) difference between Arabica and robusta to rising CO_2_ with respect to the degree of [CO_2_] stimulation. Such divergence is evident in leaf weight, number of branches, and above ground biomass (Fig. 4).

In addition to differential growth response to rising [CO_2_], numerous reports have indicated CO_2_ induced changes in secondary plant chemistry^4^. Of ubiquitous note in these observed changes is the CO_2_ induced decline in protein and N, with subsequent increases in C:N ratio^26^. In the current study, similar N declines were observed, but no interspecific or intraspecific differences were recorded. However, caffeine concentration (mg g^−1^) when averaged for all Arabica cultivars and for robusta combined, declined with additional [CO_2_], and this decline was significantly more for robusta vs. Arabica. Whether such declines may improve or reduce beverage quality in the future remains to be determined.

If caffeine acts as a deterrent against herbivores^27–29^, a reduction in caffeine in coffee leaves and seeds might result in decreased ability against deterrence, and consequently, increase pest pressure on the plants. Even though the projected effects of climate change on the coffee berry borer (*Hypothenemus hampei*), coffee leaf miner (*Leucoptera coffeella*), coffee white stem borer (*Monochamus leoconotus*), root-knot nematode (*Meloidogyne incognita*), and coffee leaf rust have been examined^38^, none of these studies considers possible changes in caffeine levels, and other chemistry, as a result of increasing CO_2_ levels.

The results presented here indicate no significant intraspecific variation in response to [CO_2_] among Arabica cultivars and hence, no clear indication as to whether recent or projected changes in atmospheric CO_2_ could be used as a selection factor for Arabica coffee adaptation. However, there appear to be clear interspecific differences between Arabica and robusta in relation to both growth and caffeine concentration at [CO_2_] above a 300 ppm baseline. Such differences suggest potential for differential selection in fitness as CO_2_ continues to increase.

There are some obvious challenges in analyzing these data in the larger context of whether recent or projected [CO_2_] can be used to select for more CO_2_ responsive coffee cultivars or coffee species. For example, vegetative development is known to be the most sensitive stage of growth in relation to rising [CO_2_]^23^ and has been suggested as a means to select for cultivar responsiveness in annual crops^9,39^. However, for tree crops, with slower relative growth, first year assessments may be useful in assessing initial response, but insufficient to discern longer-term differential effects on seed production (i.e., crop yield and quality). There are additional interspecific and intraspecific issues related to environmental shifts likely to change in parallel to rising [CO_2_] such as precipitation and/or temperature that, in turn, will also influence selection and adaptation of coffee to climate change. Yet, as indicated by these initial data, it seems unlikely that Arabica and robusta will respond similarly to increasing [CO_2_] and such potential differences may have long-term qualitative and quantitative consequences for Arabica and robusta production globally. In addition, it will be of interest to compare interspecific differences between Arabica with *C. eugenioides*, the other parent of Arabica coffee in a future study to determine if a similar response pattern is observed for *C. eugenioides*.

The basis for differential responses to rising [CO_2_] between Arabica and robusta is uncertain. They may be related to: (1) interspecific variation, due to physical (morphological) or physiological differences, or a combination of both; (2) the effect of polyploidy, which amongst other features, influences cell size, genomic stability, gene expression and evolution rates^40^. All species of coffee are diploid (2n = 2x = 22), except Arabica coffee, which is an allotetraploid (2n = 4x = 44)^41,42^. One of the recorded features of polyploidy in coffee is that higher ploidy results in fewer but larger stomata^43,44^, and this may be linked to the different CO_2_ effects we record in coffee. Another well-known consequence of polyploidy and specifically allopolyploids, is self-compatibility (self-fertilization)^45–47^. There are numerous evolutionary consequences for self-compatibility, including the reduction in genetic diversity^48^; for cultivated (farmed) Arabica coffee, this would be compounded by the severe genetic bottleneck created through the domestication process^49^. This may explain the lack of difference in CO_2_ response in the three Arabica cultivars we have examined, although our sample size is not large enough to make any meaningful assessment.

There would appear to be potential for [CO_2_] to be used as a selective factor in adaptation and yield response for tree and perennial crops. Such efforts, however, are still in their infancy. The current study, the first to examine Arabica and robusta responses to recent and projected levels of CO_2_, from germination through the first year of growth, is suggestive of either interspecific differences or polyploidy level, but additional, long-term information will be needed to adequately determine how, and to what extent, recent and ongoing increases in [CO_2_] and/or climate change may act as a selection factor among Arabica cultivars. Moreover, it will be necessary to consider drought stress (reduced water availability), which so far has received scant attention in CO_2_ enrichment influences for coffee with regard to climate change^50^. It has been argued that the influence of climate change on coffee production has been overestimated, although work so far has focused on elevated air temperatures^22^. Indeed, mitigation of elevated temperatures due to elevated CO_2_ does seem to offer potential where there is adequate soil water availability (e.g., at field capacity)^19,20^ but in many circumstances it is soil water availability (including temporal availability), and its relationship with other climatic variables (including temperature), that is the main limiting factor when considering climate change induced morbidity and mortality^50^. The interaction between elevated CO_2_ and abscisic acid signaling, stomatal closure and CO_2_ influx_,_ as well as other physiological and chemical processes involved with drought^51^, require careful investigation.

## Methods

### Seeds

Three Arabica cultivars widely grown throughout Latin America were tested: cv. ‘Bourbon’, cv. ‘Catimor’, and cv. ‘Typica’^52,53^. Typica and Bourbon are the progenitors of most Arabica coffee cultivars grown worldwide and are believed to have originated from coffee grown in Yemen of Ethiopian origin^54,55^. Arabica coffee grown in Indonesia originated from Yemen, and seeds taken from Java (Indonesia) to Amsterdam and then to the American continent led to the denomination Typica^53^. Seeds taken from Yemen and grown in Île de Bourbon (Bourbon Island; present day La Réunion) led to the denomination Bourbon^18^. Catimor is the result of crossing of two coffee cultivars: cv. ‘Caturra’ and cv. ‘Híbrido de Timor’ or ‘Timor Hybrid’ (a natural polyploid hybrid originating in Timor, an island in the Malay Archipelago, and resulting from a crossing between Arabica and robusta). Híbrido de Timor and the derived Catimor are resistant to coffee leaf rust (*Hemileia vastatrix*) and gained their resistance genes from robusta coffee^52,53^.

Mature coffee fruits for the Arabica cultivars were collected in August 2016, and again in September 2017 from plants at Rancho El Porvenir (869 masl; N 15.13229, W 92.20151) in Chiapas, Mexico. Robusta has higher levels of caffeine compared to Arabica (ca. 1.7% vs. 1%, respectively)^56^ and is adapted to growth at lower elevations in Guineo-Congolian forests^57^ and thus warmer and mostly wetter conditions relative to Arabica, which originates from high altitudes forest in Ethiopia and South Sudan and is adapted to a cooler, more seasonal environment^58^. Robusta fruits were collected in 2016 and again in 2017 from plants at Ejido Salvador Urbina (693 masl; N 15.04415 W 92.18578) in Chiapas, Mexico. Fruits were depulped, fermented, washed, and dried (ca. 12% moisture) and sent to the USDA-ARS Beltsville laboratory for germination.

### Planting

Twelve plastic bins measuring ca. 60 cm × 50 cm × 33 cm deep (ca. 99 L by volume) were used to provide three monocultures of the four (three Arabica and one robusta) taxa for each [CO_2_] treatment (four chambers). Each bin was perforated with 12 holes (1 cm diam.) to allow for water drainage. A screen mesh was placed at the bottom of each bin prior to adding the growing medium (Pro-Mix BX; Premier Horticulture Inc., Quakertown, CA, USA) to minimize growing medium loss after watering.

Seeds were soaked in water 24 h prior to planting, to promote germination. Each bin was moistened before planting 72 seeds per tub, ca. 2.5 cm deep, and ca. 5 cm apart. For the first run, seeds were planted on August 10, 2016 and the first germination occurred on September 5, 2016. For the second run, seeds were planted on September 12, 2017 and the first germination occurred on October 11, 2017. Rates of germination did not vary as a function of [CO_2_].

For both trials, nutrients were initially provided at sowing and again at two months post-planting using a complete nutrient solution^59^. MiracleGro 24-8-16 (Marysville, OH) was provided at ca. 3 months following planting and given at 2–3 weeks’ intervals until final harvest. An iron chelate micronutrient (Sprint 330, Becker Underwood, Ames, IA, USA) was sprayed as needed. The growth medium/soil was maintained at, or close to, field capacity.

### Environmental chambers

Providing pre-ambient [CO_2_] concentrations is not possible *in situ*; therefore, controlled environment chambers (Bio-Chambers, Incorporated, Winnipeg, Canada) were used. The temperature for each chamber was kept constant at 25 °C, day/night. Light, quantified as photosynthetically active radiation (PAR), was maintained at 400 µmol mol^−1^. The daily light period was 12 h light was supplied by height-adjustable, dimmable banks of metal halide and high-pressure sodium bulbs (400 µmol m^−2^ s^−1^).

CO_2_ concentrations were maintained by injection of either CO_2_ or CO_2_-free air using a TC-2 controller that monitors [CO_2_] in real time as measured by an infrared gas maintained in absolute mode. To maintain a range of recent and projected atmospheric CO_2_, concentrations were set at 300, 400, 500 and 600 ppm, 24 h day^−1^. These [CO_2_] values represent the measured Mauna Loa values from 1915 to 2015, and those projected by the end of the current century^60^. Actual mean [CO_2_] values (+SD, in [ppm]), from measurements recorded every three minutes throughout the experiments in each of the chambers, were 326 ± 38.6, 430 ± 42.7, 511 ± 26.2, and 607 ± 27.9 in the first run, and 303 ± 23.2, 409 ± 29.6, 499 ± 20.4, and 596 ± 23.0 in the second run.

### Harvests

Destructive harvests were performed at three different times, ca. 4, 7, and 12 months post-planting. At each harvest, 3–5 plants within a bin (for all taxa and [CO_2_] treatments) were removed from the tubs, height determined (cm), then separated into leaf laminae, branches, stems, and roots. Leaf (cm^2^) area was determined photometrically using a leaf area meter (Li-Cor 3100, Lincoln, NE, USA). All plant material was weighed (g) after drying at 65 °C until dry weight was constant. Root binding did not occur as indicated by visual examination at the conclusion of the experiment when plants were removed from tubs.

### C:N ratios and caffeine analysis

For each sample, all leaves, per plant were pooled and oven-dried (65 °C) until the sample was completely dry. Each dried sample was ground using a Wiley Mill with a mesh size #20. Total C and N contents were determined using a Vario Max CN (Elementary Americas, Inc., Ronkonkoma, NY, USA). Nitrogen and carbon content were determined as a percentage of the dry weight of the sample.

For extraction and determination of caffeine, leaves within a replicate were flash frozen in liquid N and stored at −80 °C until lyophilized. Leaves were then pulverized using an A11 Basic Analytical Mill (IKA Works Inc., Wilmington, NC, USA). A total of 100 mg of pulverized leaf material was added into 15 ml centrifuge tubes with 5.0 mL of a 70% methanol/water mixture. Tubes were then vortexed for 30 s and sonicated for 60 min. The slurry was centrifuged at 5,000 rpm for 10 min before being diluted (1:20), filtered, and ultimately stored in 1.5 mL HPLC vials. All reagents used for the analysis were of HPLC grade purity and prepared fresh on each day of the analysis. Instrumental analysis was performed using a Shimadzu Prominence High Performance Liquid Chromatograph (Shimadzu Scientific Instruments, Columbia, MD, USA) using a mobile phase of 80% methanol/water and 15 mM phosphate buffer at pH 6.2. Separation was conducted using a Thermo Scientific Aquasil reverse phase C18 column (4.6 × 250 mm, 5 µm particle size; Thermo Fisher Scientific, Waltham, MA, USA) at a flow rate of 0.550 ml/min. Detection and quantification was done using a UV detector at 275 nm and determined using a calibration curve. The caffeine calibration curve was created using an HPLC grade caffeine standard (99.7% purity; ACROS Organics #10816-5000; Thermo Fisher Scientific, Waltham, MA, USA) across five concentrations 2.5, 5, 10, 20, and 25 ppm. The fitted curve showed excellent linear responsivity as demonstrated by an r^2^ of 0.998. In addition, there was negligible variation between replicate injections at 10 ppm using the same standard as measured by its percent relative standard deviation of 0.385%.

The caffeine concentration in leaves can also be used as a proxy for concentrations in coffee beans, based on a correlation between caffeine concentration in seedling leaves and seeds^61,62^. Dias Chaves *et al*.^61^ focused on the 1^st^ and 3^rd^ pair of leaves in the seedlings, while de Moraes *et al*.^62^ used the 3^rd^ and 4^th^ pair. We found no significant differences in caffeine content between the last pair of fully expanded leaves and all remaining leaves combined (cotyledons excluded; using March 2017 samples, i.e., first year, second sampling; 7 months and 18 days post-planting). Based on these results, we pooled all leaves at each sampling date for caffeine analysis. Mazzafera and Magalhães^63^ found no correlation between leaves and seeds, but these were collected from mature plants, not seedlings.

### Statistical analysis

Three replicate bins for each Arabica cultivar and for robusta coffee (i.e., 12 bins per chamber) were present for each of four [CO_2_] treatments. Within each chamber [CO_2_], bins were randomized; and randomized again after the first two harvests at 4 and 7 months to avoid edge effects. After the first run of the experiment (i.e., one year), the chambers were randomly reassigned [CO_2_] treatments and the experiment repeated. Humidity, PAR, and temperature were quantified before and at the end of each harvest to determine within chamber and among chamber variability. Values for each parameter were consistent between experimental runs. All measured parameters were based on tub averages (3–4 plants per tub) for both runs. All measured and calculated parameters were analyzed using analysis of variance including [CO_2_], Arabica cultivars, Arabica vs. robusta, and harvest time (Statview Software, Cary, NC, USA).
